# End-of-life care among Koreans in critical care and community-dwelling Korean Americans: A cross-cultural scoping review

**DOI:** 10.1017/S1478951525101090

**Published:** 2025-12-05

**Authors:** Soo Hyun Kim, Changhwan Kim, Erh-Chi Hsu, Zackary Berger, Hae-Ra Han, Binu Koirala, Jung Kwak, Katherine A. Ornstein, Rebecca Wright

**Affiliations:** 1 Johns Hopkins School of Nursing, Baltimore, MD, USA; 2 Johns Hopkins School of Medicine, Johns Hopkins Berman Institute of Bioethics, Baltimore, MD, USA; 3 The University of Texas at Austin, School of Nursing, Austin, TX, USA

**Keywords:** End of life, palliative care, cross-cultural research, Korean, Korean American

## Abstract

**Objective:**

End-of-life (EOL) care for critically ill individuals is shaped by socioeconomic, legal, and cultural factors for Koreans in South Korea and Korean Americans (KA) in the United States. This scoping review thematically synthesized critical care literature from Korea and community-based literature involving KAs to inform culturally tailored EOL and palliative care research and practice.

**Methods:**

Following the updated JBI scoping review guidance, we reviewed English and Korean articles across seven databases. Due to the lack of critical care studies involving KAs, the scope of U.S. studies was broadened to all healthcare settings. We conducted a thematic synthesis to identify cross-context cultural insights that are potentially transferable from Koreans in critical care to KAs with similar needs.

**Results:**

Evidence on EOL care for Koreans in critical care and for KA communities across U.S. settings was limited. Korea-based critical care studies (*N* = 23) highlighted physician-initiated decision-making, minimal advance care planning, and a lack of direct patient perspectives. U.S.-based studies (*N* = 26) focused on hypothetical palliative care preferences among older, community-dwelling KAs, with limited attention to critical care. Both contexts revealed shared cultural preferences for family-centered decision-making, physician-led discussions, and indirect communication about diagnosis and prognosis. Further research is warranted to investigate within-group heterogeneity and preference shifts across illness trajectories to inform culturally tailored EOL interventions for KAs.

**Significance of results:**

Findings highlight the need for culturally and structurally informed approaches to improve EOL care in both Korea and the U.S. This cross-context analysis demonstrates how evidence from the heritage country can inform research and practice for immigrant and minoritized populations when domestic data are sparse. Strength-based approaches grounded in community values, combined with culturally specific insights from Korean literature, may enhance culturally responsive support for KA patients and families.

## Introduction

End-of-life (EOL) communication and decision-making have become central to intensive care unit (ICU) care due to technological advances and increasing survival rates (Curtis et al. 2022). Aligning care goals with patient and family values is vital, yet transitioning to palliative care in times of critical illness remains challenging due to emotional, ethical, cultural, and psychosocial complexities (Coombs et al. 2012). These challenges are shaped by broader socioeconomic, legal, and cultural factors (Kwak and Haley 2005; Wang et al. 2018), which must be addressed to ensure comprehensive support.

This review focuses on Korean American (KA) populations, broadly defined to include United States (US)-born individuals of Korean heritage, Korean individuals with naturalized citizenship, and recent immigrants from South Korea. KA populations represent the fifth-largest Asian group, now numbering nearly two million – a 17% increase over the past decade – in the US (U.S. Census Bureau 2022), which is home to the largest Korean diaspora outside of Korea (Esterline and Batalova 2022). Despite this growth, their experiences with EOL care remain poorly understood. Available research revealed that Asian Americans – one of the fastest-growing and most ethnically diverse populations – receive more intensive treatments at EOL, die in hospitals frequently, and are less likely to use hospice or palliative care than non-Hispanic White Americans (Jia et al. 2022; Lackan et al. 2009; Ngo-Metzger et al. 2008). However, these findings often obscure subgroup differences by collapsing over 40 distinct ethnicities under a single “Asian and Pacific Islander” umbrella – an increasingly criticized practice masking cultural and healthcare differences (Holland and Palaniappan 2012; Jin 2021).

KA populations face unique challenges navigating the US healthcare system, where traditional family-centered and relational values from Korea may conflict with or adapt to more individualistic norms in the US (Jang et al. 2020). Individuals within this population often seek support from their ethnic communities and culturally concordant providers, as their challenges are compounded by the complexities of immigration status and navigating an unfamiliar system (Choi 2013). These challenges highlight the critical need for culturally tailored care for KA populations. However, existing research has focused on community settings (Park and Hendrix 2018), leaving a gap in our understanding of how KAs engage in EOL care in hospital contexts.

To address the gap in the critical care literature on KA populations, we conducted a cross-context scoping review that maps ICU-based EOL literature in Korea alongside EOL literature involving KAs in the US to identify transferable cultural implications. We prioritized ICU-based studies from Korea that provide relevant contexts for US clinicians and policymakers by clearly illustrating how cultural values influence EOL decision-making during high-acuity care and nascent ICU palliative care models. With ongoing migration from South Korea to the US (Esterline and Batalova 2022), evidence from Korea may inform culturally responsive care and policymaking for the KA population with critical illness. Further, exploring Korean literature in the context of recent legislation of the Hospice, Palliative Care, and Life-Sustaining Treatment Act (LST Act) in Korea (Choi et al. 2019) and the development of ICU-based palliative care is important, as approximately 75% of Koreans die at medical institutions with persistent unmet EOL needs (Cheon et al. 2023). This cross-cultural exploration aims to add nuance to the current understanding of Korean cultural values, which are often associated with Confucian principles wherein filial piety, emphasizing respect for parents and elders, may discourage open discussions about death or treatment withdrawal (Choi et al. 2023; Lee et al. 2021).

As healthcare practices evolve globally, cross-cultural influences on palliative care will likely increase (Rosa et al. 2018). Prior reviews have examined EOL care among KA individuals (Park and Hendrix 2018; Suk et al. 2021), but none have synthesized Korean and American EOL literature together to guide culturally responsive palliative care for KA populations. Furthermore, no review to date has mapped the literature on ICU-based EOL care in Korea. Therefore, this review aimed to (1) map the current state of science on EOL care for critically ill Korean individuals and all KA individuals in the US, and (2) offer culturally informed directions for future research and practice in both countries.

## Method

Our scoping review follows the updated JBI methodological guidance and the Preferred Reporting Items for Systematic Reviews and Meta-Analyses (PRISMA) extension for Scoping Review (Peters et al. 2021; Tricco et al. 2018). We chose this method for the exploratory design of this review, aiming to guide future research by mapping the literature.

### Review question

Our review questions are structured using the “Patient-Concept-Context” framework (Peters et al. 2021): Patient – Koreans, KAs, Korean immigrants; Concept – EOL and palliative care; Context – Critical care in Korea and all settings in the US.

1. What is known about EOL care for critically ill Korean individuals in Korea and among KA communities – including US-born, naturalized US citizens, and immigrants – in all US settings?
2. What culturally relevant insights can inform future research and practice to improve EOL care for these populations?

### Literature search

**Figure 1.**
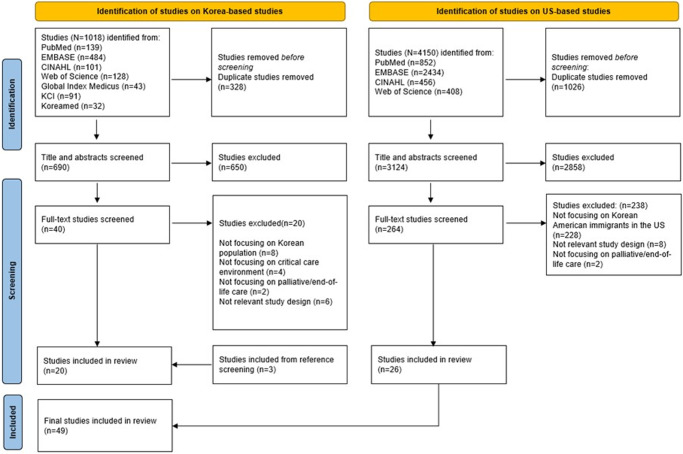
PRISMA flowchart diagram of the study.

Figure 1 shows the screening process guided by the PRISMA. A two-stage literature search was conducted to identify studies on EOL care for critically ill individuals with Korean heritage and KA populations in all US settings.

In the first stage, we focused on critical care. Seven databases (PubMed, Cumulative Index to Nursing and Allied Health Literature [CINAHL], Excerpta Medica Database [EMBASE], Web of Science, Korea Citation Index, Global Index Medicus, and KoreaMed) were searched using the terms “Korean AND EOL/palliative care AND critical care.” English and Korean studies with no time restriction were included. We excluded studies focusing on pediatrics, studies not addressing patients and families (e.g., providers’ moral distress), studies outside critical care settings, and studies lacking discrete data on Koreans). Unpublished dissertations, conference proceedings, editorials, letters, and review papers were also excluded. Search strategies were validated by a university informaticist (see Appendix A). Three authors (SHK, CK, and ECH) screened the studies, resolving conflicts by consensus. Of 1018 records, we removed 328 duplicates. From 40 full-text articles and three additional studies identified from references, we included 23 studies, all conducted in Korea. None addressed KAs in critical care.

Due to this gap in KA populations, we expanded the focus to include EOL and palliative care for KAs in any care environment. Using the search terms “Korean AND EOL/palliative care” with a US geographical filter, we searched four databases (PubMed, CINAHL, EMBASE, Web of Science). From 4150 records identified, 1026 duplicates were removed. After abstract and full-text reviews, 26 US-based studies were included. Taken together, our final sample included 49 studies.

### Data extraction & analysis

We conducted a thematic synthesis to identify transferable cross-pollination of cultural insights across Koreans in ICUs and KA populations in US settings (Thomas and Harden 2008). We juxtaposed and synthesized two literature sets to identify cultural values and patterns in EOL care that are relevant to research and care models for the KA population while mapping both literatures’ scopes and gaps. This approach aimed to conceptually translate rather than compare, as care settings and systems were not equal or directly comparable. Findings were interpreted while accounting for differences in care acuity, decisional capacity, and structural factors. Data were initially charted into three tables: Table 1 summarizes general characteristics, while Tables 2 and 3 present detailed findings. We then inductively developed thematic categories through iterative reading and open coding of the extracted data and the original authors’ interpretations (Thomas and Harden 2008). One author (SHK) conducted data extraction, coding, and thematic development, and the work was reviewed and validated by three co-authors (CK, ECH, RW).

**Table 1.** General characteristics of the included studies

Characteristics	Categories	Number of Korean studies (%)	Number of US studies (%)
**Publication year**	−2000	0	4 (15%)
	2000–2009	2 (9%)	5 (19%)
	2010–2020	11 (48%)	13 (50%)
	2020–	10 (43%)	4 (15%)
**Study design**	Quantitative		
	Cross-sectional survey	7 (30%)	15 (58%)
	Prospective survey	2 (9%)	0
	Retrospective cross-sectional	5 (22%)	0
	Retrospective cohort	5 (22%)	0
	Qualitative		
	Case study	1 (4%)	1 (4%)
	Community-based forums	0	1 (4%)
	Ethnography	1 (4%)	1 (4%)
	Grounded theory	0	4 (15%)
	Descriptive/Exploratory design	2 (9%)	2 (8%)
	Mixed method	0	1 (4%)
	Quality improvement trial	0	1 (4%)
**Sample size^a^** (Korean ethnic individuals)	<50	5 (22%)	10 (38%)
	50–99	7 (30%)	2 (8%)
	100–499	8 (35%)	11 (42%)
	500-	3^b^ (13%)	2 (8%)
**Participants of interest**^c^	ICU patients/older adults	9^b^ (39%)	22 (85%)
	Family caregivers (including surrogate reports)	8 (35%)	4 (15%)
	Healthcare providers (including surrogate reports)	8 (35%)	1 (4%)

a One US-based study did not report sample size.

b Only retrospective designs were used.

c This was counted, allowing for overlaps between categories.

**Table 2.** Detailed characteristics of Korea-based studies

Author, year	Study design	Sample	Key variables/Interview questions	Findings
Byun et al. 2003	Quantitative, cross-sectional design	*N* = 101 nurses, 88 family members Mean age not reported	Survey: Attitude/Attitudes toward WWLT	84.2% of nurses and 73.9% of families agreed with the necessity of WWLT, with the reason of ‘loss of meaning of life’ for irreversibly critically ill patients.
Lee et al. 2008	Quantitative, retrospective cross-sectional design	*N* = medical records of 102 deceased ICU patients Mean age: 56.6	Medical record: Frequency of DNR order/violated DNR orders, Timing of death after DNR order	Among the 73.5% of patients with DNR orders, 96% of DNR were suggested by physicians. 84% of patients with DNR orders died within 3 days of order consent. The DNR order was violated in 12% of the cases.
Lee and Kang 2010	Quantitative, cross-sectional design	*N* = 80 nurses, 80 physicians, 80 family members Mean age not reported	Survey: Awareness/Attitudes towards WWLT	Over 90% of ICU nurses, physicians, and families agreed with the necessity for WWLT. Three groups responded the most negatively toward utilizing all possible treatments to extend patients’ lives. Three groups placed the biggest decision-making authority with the patients.
Shin et al. 2014	Quantitative, retrospective cross-sectional design	*N* = medical records of 89 deceased ICU patients Mean age: 65.8	Medical record: Frequency of WWLT, AD, DNR decisions, family discussion, Timing of death after WWLT decision	In 63 out of 64 WWLT cases, discussions with families were initiated by physicians when a patient was expected to die soon. No patient had completed AD before or during ICU admission. Patients died on average 2.7 days after the WWLT decision. Death within one day of the consent occurred in 51.7% of the cases and was more common in those with verbal consent.
Kim et al. 2015	Quantitative, cross-sectional design	*N* = 76 family members and 91 ICU nurses Mean age not reported	Survey: Awareness toward WWLT	Family members preferred to be informed about the need for a DNR when spontaneous breathing has stopped or in the event of a coma. Regardless of the patient’s ability to express their wishes, family members were less likely than nurses to perceive the necessity of DNR and treatment discontinuation (*P* < 0.001).
Baek et al. 2016	Quantitative, retrospective cross-sectional design	*N* = medical records of 80 patients with DNR orders Mean age: 64.5	Medical record: Timing of DNR orders, Medical management outcomes, ICU length of stay	Those with DNR orders after 48 hours of admission (Late DNR group) stayed longer in ICU than those with DNR orders within 48 hours (early DNR group) (*P* < 0.001). Medical management was not different for the two groups except that the late DNR group had a higher rate of tracheostomy. All DNR consents were written by family members, mostly sons and daughters.
Kim et al. 2016	Quantitative, retrospective cohort design	*N* = 45 ICU patients (total 125 acutely ill patients aged over 80 referred to ICU) Mean age: 84.7	Medical record: Frequency and timing of DNR, relationships, and the number of those in decision-making	A greater number of family members in the decision-making was associated with intensive care initiation (*P* < 0.05). Only 20% (9/45) of patients had DNR decisions before ICU referral; of these, 82.9% (8/9) were made by offspring. 51.1% (23/45) of the family members of patients admitted to the ICU later wanted DNR. Among them, 16 out of 23 patients agreed to the decision during ICU stay, and 7 agreed after ICU discharge.
Min et al. 2016 (32)	Qualitative, case study	*N* = 8 meetings with families of 8 patients Mean age not reported	Context, process, and outcomes of the implemented family meetings	Family meetings were held on average 11.8 days after ICU admission. 7 out of 8 family meetings came to a decision of WWLT, DNR, or full active treatment. Family meetings guideline was modified based on outcome and feedback. Meeting to be offered on demand, role expansion of other ICU staff than doctors, and providing informational materials to families were suggested.
Koh et al. 2017	Qualitative, descriptive design	*N* = 50 ICU nurses Mean age not reported	Interview: Perception towards LST in ICU at EOL	Negative views of nurses on LST in ICUs focused on suffering and pain from invasive procedures, loss of patient dignity, limited family time, patient/family regret over decisions, and the financial and emotional burden. Positive views were related to patients’ strong will to survive and families’ sense of filial duty to do everything possible.
Cho et al.^2019^	Quantitative, cross-sectional design with an additional survey after the LST Act	*N* = 255 deceased ICU patients (reported by 177 medical staff) Mean age: 66.6	Survey: QODD reported by staff within 48 hours of patients’ death	Mean score of QODD was lower than previous U.S. or Dutch studies. QODD was positively correlated with receiving analgesics (*P* = 0.003) and was negatively correlated with receiving CPR (*P* < 0.001) and inotropes (*P* = 0.008) within 24 hours before death. QODD after establishment of LST Act showed tendency to increase (31.3% to 35%, *P* = 0.087).
Choi et al. 2019	Quantitative, cross-sectional design	*N* = 16 ICU family members (and 23 hospice family members) Median age: 82 (decedent), 52 (family)	Survey: QODD reported by surrogates	Mean score of QODD reported by ICU family members was lower than that of the hospice group (p = 0.03). The ICU group rated lower on “pain under control,” “breathing comfort,” and “dignity and self-respect.” A subgroup who rated higher QODD on “preparation for death” included patients who transitioned to general wards before death.
Jo et al. 2019	Quantitative, cross-sectional design	*N* = 71 adult family members Mean age: 63.8 (patients), 49.6 (family)	Survey: QOC, Anxiety, Depression, Post-traumatic stress	Family members perceived the QOC with providers regarding patients’ impending death, spiritual or religious beliefs, and patients’ treatment preferences as very low and often not performed. Family members’ depression showed a weak, negative relationship with QOC with nurses (*P* = 0.03).
Lee et al. 2019	Quantitative, retrospective cohort design	*N* = 56 homeless ICU patients and 112 non-homeless ICU patients Mean age: 54.4 (homeless); 54.0 (non-homeless)	Medical record: Procedure before death, Frequency of EOL decision-making by family	Homeless ICU patients were significantly less likely than non-homeless ICU patients to have family or substitute decision-makers (*P* = 0.03). A larger proportion of homeless ICU patients died after cardiopulmonary resuscitation than non-homeless ICU patients (*P* = 0.005).
Lee et al. 2020	Quantitative, retrospective cross-sectional design	*N* = medical records of 80 ICU patients (total 227 patients) Mean age: 72.5	Medical record: Frequency of WWLT, ICU length of stay, Type/Legal form of WWLT decision	6.3% of cases completed AD in the ICU. 40% of ICU patients withdrew and withheld all treatments in the ICU. There were more cases LST withheld than withdrawn (76.3% vs. 23.8%). 65% of decisions were made by agreement of all families as surrogates. Higher proportions of treatment withdrawal and ICU-to-ward transfers were observed in cases with intensivist involvement compared to those without (50.0% vs. 4.3% and 52.9% vs. 19.6%, respectively).
Oak and Kim 2020	Quantitative, cross-sectional design	*N* = 114 family members Mean age: 47.3	Survey: Attitude toward death, Perceptions of hospice care, Hospice care needs	The item with the highest score for attitudes toward death was, “The idea of life after death bothers me greatly.” Minimizing pain was perceived as the most critical hospice care need. Family members with better awareness of hospice care had greater hospice care needs (*P* < 0.001).
Kim and Tak 2021	Quantitative, cross-sectional design	*N* = 89 family members Mean age not reported	Survey: Knowledge of LST, Attitude toward LST, Awareness of the 2018 LST Act	Family members’ knowledge of LST was a predictor of their attitude (*P* = 0.021). 73% of participants were aware of the 2018 LST Act. Knowledge of LST (*P* < 0.001) and attitude toward LST (*P* = 0.03) positively correlated with the awareness of the LST Act.
Lee et al. 2021	Quantitative, retrospective cross-sectional design	*N* = health insurance data of 19,119 deceased patients aged 65 and older in the ICU Mean age at death: 76.6	Health insurance data: Frequency of high-intensity care use at end-of-life in ICU	Transfusion was the most common high-intensity care procedure (68.9%), followed by mechanical ventilation (50.6%) and hemodialysis (35.7%). Patterns of high-intensity care differed based on the primary cause of death. Those who are younger and those with longer hospitalization were likely to use high-intensity care (*P* < 0.001). Annual cost of high intensity of care steadily increased during 2016–2019.
Lee et al. 2022b	Quantitative, prospective longitudinal design	*N* = 252 deceased patients (reported by 143 staff) Mean age: 68.6	Survey: QODD reported by staff within 48 hours of patients’ death (compared with Cho et al. (2019))	Implementation of the LST Act was positively associated with quality of death in ICU, with an increase in QODD scores compared to the findings of Cho et al. (2019) prior to the law’s enactment (*P* = 0.001). Fewer patients were admitted to the ICU for post-resuscitation care (*P* = 0.003). Time from DNR to death became longer (*P* = 0.02). But scores on patient autonomy and emotional support were still low.
Choi et al. 2023	Qualitative, focused ethnography	*N* = 23 nurses, 10 physicians, 4 family members Mean age not reported	Interview: Perception of WWLT experiences, Power dynamic of decision-making among providers and family members	Family members were empowered in LST decisions due to legal requirements, familism culture, filial duty, and superiority in the customer-provider relationship. Those who pay the medical cost, were closer to patients or the firstborn commonly become the primary decision-makers. WWLT decisions were shaped by families’ perceptions of the illness, physicians’ explanation of treatment futility, desire for patients’ comfortable death, and financial burden. Providers and families shared the goal of preserving patients’ dignity.
Jang et al. 2023	Quantitative, retrospective cohort design	*N* = 89 patients who died in ICU (total 255 patients who died in wards and ICUs) Mean age (total patient sample): 61.6	Medical record: Frequency of WWLT decisions, Documents used for WWLT decisions, Main decision-makers	Patients’ participation in WWLT decision was lower in the ICU than in the wards (20.6% vs. 79.4%) More ICU patients had POLST documentation based on family consensus compared to ward patients (51.7% vs. 30.7%). A higher percentage of ICU patients wrote ADs compared to those in the ward (15.7% vs 5.4%).
Chang et al. 2024	Qualitative, descriptive design	*N* = 20 ICU nurses Mean age: 30.1	Interview: Nurses’ position in caring for ICU patients undergoing WWLT, and how their death is addressed in the system	ICU nurses observed no regulations or guidelines about end-of-life care for patients undergoing WWLT, with organizational culture, managers, and physicians pressuring them to prioritize patients requiring intensive care. ICU nurses observed that patients and their families rarely discuss death before making WWLT decisions, and conversations about dying or WWLT process were seldom initiated until the patient was no longer capable of participating.
Kim et al. 2024	Quantitative, retrospective cohort design	*N* = medical records of 238 ICU patients who withdrew LST and a control group of 513 who didn’t Median age: 78 (withdrew), 75 (control)	Medical record: Types of intensive care, Days from admission to LST withdrawal or death, Main decision-maker (patient or family)	Older age and higher readmission rates were significantly associated with LST withdrawal in the ICU (*P* = 0.002, *P* = 0.01). The average time of LST withdrawal to death was 2 days. Patients with infectious disorders were twice as likely not to withdraw from LST compared to cancer and neurological patients (*P* = 0.001). In 86% of withdrawal cases, family members made withdrawal decisions. 30% of cancer patients made their own decisions, compared to 11% and 5% among those with neurological and infectious diseases, respectively.
Lee et al. 2024	Quantitative, retrospective cohort design	*N* = medical records of 577 ICU patients Mean age: 69.5	Medical record: DNR/POLST documentation, Treatments received in ICU, mortality, ICU length of stay	36.6% completed DNR/POLST, with 22.7% of them having completed before ICU admission. Those who had DNR/POLST received more invasive treatments and mechanical ventilation than those without DNR/POLST (*P* < 0.001). Those with DNR/POLST had higher ICU and in-hospital mortality and longer stays in ICU (*P* < 0.001).

*Notes*. AD: advanced directives, DNR: do not resuscitate, ICU: intensive care unit, LST: life-sustaining treatment, LST Act: Hospice, Palliative Care, and Life-Sustaining Treatment Act, N: number, POLST: physician order of life-sustaining treatment, WWLT: withhold or withdraw life-sustaining treatment, QOC: quality of communication, QODD: quality of death and dying.

**Table 3.** Detailed characteristics of US-based studies

Author & year	Study design	Sample	Setting	Key variables/Interview questions	Findings
Blackhall et al. 1995	Quantitative, cross-sectional design	*N* = 200 KAs aged 65 and older (with 600 Mexican/ African/ European Americans) Age, immigration information not reported	31 senior citizen centers in Los Angeles, California	Survey: Ethnic differences in attitudes toward patient autonomy	KA participants were the least supportive of truth-telling about terminal illness. 60% believed that the family not the patient should decide on using LST. Higher education levels correlated with preferring autonomous decision-making on using LST (*P* < 0.05). Younger individuals were more likely to want to be informed of their diagnosis (*P* < 0.01).
Murphy et al. 1996	Quantitative, cross-sectional design	*N* = 200 KAs aged 65 and older (with 600 Mexican/ African/ European Americans) Age, immigration information not reported	31 senior citizen centers in Los Angeles, California	Survey: Knowledge of, attitude towards, and possession of AD	13% of KA participants had knowledge of AD. KA participants were more likely to have a negative attitude toward ACP than African and European Americans (*P* < 0.0001). No KA participants had written ADs.
Frank et al. 1998	Qualitative, case-study interview, narrative approach	*N* = a typical KA participant who explained a contradiction in Blackhall et al. (1995) Age: 79 A first-generation immigrant	A senior citizen center in Los Angeles, California	Interview: Participant’s use of Korean ethics or values in decision-making preferences and practices	Despite negative views on LST, participants felt adult-children had a filial duty to use it to prevent parents’ death. Participant believed disclosing a diagnosis could cause distress and obsessive thinking about death. Not signing AD was linked to preferring family to make decisions when time comes. The decision on place of death was influenced by the wish to avoid burdening the family.
Blackhall et al. 1999	Mixed-method, survey and ethnographic interview	*N* = 200 KAs aged 65 and older (survey), among these, 20 KAs (interview) Age, immigration information not reported	31 senior citizen centers in Los Angeles, California	Survey: Ethnic differences in attitudes toward AD Interview: General attitude and personal desire for LST	Participants had the most positive general attitude toward LST use compared to African/Mexican/European-Americans but had a low personal desire for LST (*P* < 0.0001). Participants personally prioritized their family’s wishes over their own.
Blackhall et al. 2001	Qualitative, ethnographic interview	*N* = 20 KAs aged 65 and older Age, immigration information not reported	31 senior citizen centers in Los Angeles, California	Interview: Attitude toward truth-telling	The weighing of benefits versus harms of truth-telling depended on one’s self-perception. Participants reported that those who see themselves as sick, weak, and in need of protection may view truth-telling as harmful. In “high context” cultures like Korea, truth is often conveyed indirectly, partially, or nonverbally, allowing for greater hope of living.
Phipps et al. 2003	Qualitative, community forums	Not described Age, immigration information not reported	Various KA organizations and churches in Philadelphia	Interview: Attitude and view toward ACP	Few participants knew about ACP, hospice, or their rights to make LST decisions. Some found the language in translated versions of the State Living Will and Five Wishes abrupt and frightening. Many participants struggled to discuss ACP or terminal diagnoses to shield parents or grandparents from receiving bad news. Despite this, many KAs recognized the importance of knowing one’s diagnosis and prognosis for care planning and preferred to forgo LST in terminal or vegetative states.
Kwak and Salmon 2007	Qualitative, modified grounded theory	*N* = 20 KAs aged 65 and older and 16 caregivers aged 18 to 59 caring for an older relative Mean age: 68 (older adults); 40 (caregivers) Mean years in US: 29 (older adults); 13 (caregivers)	3 counties in west central Florida	Interview: Attitudes and knowledge toward ACP and hospice, and cultural and personal expectations about EOL care.	Most participants had not heard of or misunderstood AD and hospice. Adult-children were seen as the final decision-makers, making ADs seem unnecessary, although participants expressed a need for ADs to guide their decisions. Preferences for hospice varied, with filial piety motivating both curative and palliative care. Caregivers struggled to initiate ACP conversations due to indirect communication norms and emphasized the role of physicians in facilitating EOL discussions. Participants preferred to receive information from KA physicians and Korean community organizations.
Berkman and Ko 2009	Quantitative, cross-sectional design	*N* = 26 KAs aged 65 and older Mean age: 71.2 Mean years in US: 12.7 All first-generation immigrants, primarily Korean speaking	A private primary care and two senior centers in New York City	Survey: Comfort level in EOL discussion, Preference for information disclosure, Preference about the type of information wanted	88.5% of participants were comfortable discussing EOL and believed doctors should inform them of serious illness. 57.7% preferred to avoid discussing death in advance, indicating varied preferences for disclosure timing. All participants wanted to know about palliative care. Those who had lived longer in the US, were younger, and reported better self-rated mental health were more likely to prefer disclosure of serious illness (*P* < 0.05).
Ko and Lee 2009	Quantitative, cross-sectional design	*N* = 112 KAs aged 65 and older, born in Korea and primarily spoke Korean (compared with 105 NHWs) Age not reported for KA All first-generation immigrants, primarily Korean speaking	Senior centers in New York City	Survey: EOL communication experience, ACP knowledge, Perceived susceptibility, severity, benefits, barriers related to ACP	KA participants were less likely to engage in EOL discussions compared to NHWs (*P* < 0.01). Fewer KA participants were specific about their EOL decisions compared to NHWs (6.3% < 20%). Higher ACP knowledge, perceived barriers (*P* < 0.05), perceived severity of ACP (*P* < 0.01), and more illness experience (*P* < 0.05) were stronger predictors of EOL communication engagement than ethnicity.
Berkman and Ko 2010	Qualitative, grounded theory	*N* = 25 KAs aged 65 and older, born in Korea and primarily spoke Korean Mean age: 71.2 All first-generation immigrants, primarily Korean-speaking	Two senior centers and a primary practice in New York City	Interview: Attitudes toward and preference on disclosure, timing and content of disclosure	Most participants wanted to be informed about their diagnosis and prognosis to prepare for life and death. Some preferred not to be informed fearing distress, hastening of death, and suspecting misdiagnosis. Many emphasized the importance of maintaining hope when discussing diagnosis or prognosis. Most felt that family should be informed regardless, with disclosure preferred when death was imminent.
Jang et al. 2010	Quantitative, cross-sectional design	*N* = 675 KAs aged 60 and older Mean age: 70.2 Mean years in US: 28 All first-generation immigrants	Korean organizations in Tampa and Orlando, Florida	Survey: Chronic conditions, Acculturation, Awareness of and Willingness to use hospice	73.6% of participants were willing to use hospice care. Presence of chronic conditions (*P* < 0.05), acculturation (*P* < 0.01), and prior awareness of hospice (*P* < 0.001) were significant predictors of willingness to use hospice care.
Ko and Berkman 2010	Qualitative, focus-group interview, Grounded theory approach	*N* = 23 KAs aged 60 and older Mean age: 71.2 All first-generation immigrants, primarily Korean speaking	Two senior centers and a primary practice in New York City	Interview: EOL discussion experience with their children, Perception of their children’s roles in decision making, Belief in their children to honor their wishes, Perceived influence of Korean culture	Participants faced challenges discussing EOL issues with their reluctant adult children. Some prioritized autonomy in treatment decisions, while others delegated to their children. Many preferred the eldest son as the decision-maker, though some favored collective decision-making. Confidence in their children to honor their wishes varied. Adult-children’s decisions were perceived to differ based on their upbringing and treatment futility. None of the participants had ADs. ADs were seen as helpful, though many believed their children would still opt to prolong life.
Ko and Lee 2010	Quantitative, cross-sectional design	*N* = 112 KAs aged 65 and older, born in Korea and primarily spoke Korean (compared with 105 Non-Hispanic Whites) Age not reported for KA All first-generation immigrants, primarily Korean speaking	Senior centers in New York City	Survey: ACP knowledge, Perceived susceptibility, severity, benefits, barriers about ACP	5.4% of KA participants had written ADs. Health beliefs (perceived susceptibility, severity, benefits, and barriers of ACP) were lower in KAs compared to NHWs (*P* < 0.01). Health beliefs mediated the relationship between ethnicity and AD completion. Knowledge about ACP partially mediated the relationship between ethnicity and AD completion, but only when health beliefs were excluded from the model.
Kim and Foreman 2011	Quantitative, cross-sectional design	*N* = 115 KA adult children aged 18 to 64 years Mean age: 44.5 Mean years in US: 17 First-generation immigrants: 95%	A Korean community center and three Korean churches	Survey: Life-Support Preference Questionnaire, Attitudes Toward ACP, Familism, Acculturation	Participants believed their parents would prefer a moderately high level of LST. Older age (*P* < 0.05) and higher acculturation level (*P* < 0.01) were linked to beliefs that parents would not want LST. Higher education (*P* < 0.05), acculturation (*P* < 0.01), and lower familism level (*P* < 0.05) were associated with more positive attitudes toward ACP.
Ko et al. 2012	Qualitative, grounded theory approach	*N* = 26 KAs aged 60 and older Mean age: 71.2 Mean years in US: 12.7 Primarily Korean speaking	Two senior centers in New York City	Interview: Knowledge and completion of ADs, Religiosity, Health status, Langue fluency, familiarity with and attitudes toward ADs.	None of the participants had a living will or a proxy. Half viewed a proxy unneeded since family would decide. Participants expressed lack of knowledge about ADs, need for education, and preference for community-based learning. ADs were seen as helpful for reducing decision-making burdens, but not as a guarantee of decisions. Many believed LST decisions should be made during serious illness. Cultural norms like filial piety, eldest son decision-making, collective decision-making, and focusing on life were noted.
Ko and Berkman 2012	Quantitative, cross-sectional design	*N* = 64 KAs aged 60 and older (compared with 58 Mexican Americans) Mean age: 76 Mean years in US: 23.3 All first-generation immigrants, primarily Korean speaking	5 senior facilities and a church located in an urban area on the West Coast	Survey: Attitude Toward LST, Acculturation	KA participants had less positive preferences for LST compared to Mexican Americans (*P* < 0.01). KA participants primarily spoke in Korean during interviews, had shorter US residency, and lower acculturation levels than their Mexican American counterparts.
Ko et al. 2013	Quantitative, cross-sectional design	*N* = 195 KIs aged 65 and older Mean age: 72.4 Immigration information not reported	Two senior centers in New York City	Survey: EOL discussion experience, Attitudes toward EOL discussion, Perceived burden, Number of adult children in US	21.9% of participants had experienced EOL discussions, mostly general conversations with family members. Only 11 participants had EOL discussions with a physician, social worker, or nurse. Those with more traditional Korean views on EOL (*P* < 0.01), perceived greater burden (*P* < 0.05), higher religiosity (*P* < 0.01), and more children in the US (*P* < 0.05) were less likely to engage in EOL communication.
Dobbs et al. 2015	Quantitative, cross-sectional design	*N* = 675 KAs aged 60 and older Mean age: 70 Immigration information not reported	Korean organizations in Tampa, Orlando Florida	Survey: Awareness and completion of AD, Education, Health conditions, Health insurance, Acculturation	18.3% of participants had ADs. Higher education (*P* < 0.01), more chronic conditions (*P* < 0.01), having health insurance (*P* < 0.05), and higher acculturation levels (*P* < 0.001) were positively associated with AD awareness. Acculturation predicted awareness and completion of ADs (*P* < 0.001).
Pan et al. 2015	Quantitative, cross-sectional design	*N* = 349 KA aged 18 or older Mean age: 58.5 Immigration information not reported	Various community locations in Queens County, New York	Survey: Familiarity/ Personal experience with hospice, Willingness/ Openness to hospice information	56% of KA participants were familiar with hospice. 10% of KA participants had personal experience with hospice. 87% of KA participants were willing to share hospice information with others. 74% were willing to receive hospice information.
Jang et al. 2017	Quantitative, cross-sectional design	*N* = 465 KA aged 18 or older (total 2,517 of five AA groups) Mean age: 42.8 Immigration information not reported discretely	Various community locations in Austin, Texas	Survey: AD knowledge/ completion, education level, acculturation level	Among the total sample, AD knowledge and acculturation, not education, predicted AD completion (*P* < 0.001). AD knowledge was more likely to lead to completion among highly educated individuals (*P* < 0.001). KAs had the lowest AD completion rate (5.6%) among the 5 AA groups.
Hong et al. 2019	Quantitative, cross-sectional design	*N* = 266 KA family members aged 40 or older Mean age: 54.3 First-generation immigrants: 98%	Various community locations Washington DC	Survey: Intention, Attitudes, Subjective norms. Perceived control toward ACP discussion	46% reported low ACP knowledge, and 8.5% had ADs. Positive attitudes, strong subjective norms, and high perceived control toward ACP discussions were correlated (*P* < 0.001). Intention for ACP discussion was linked to positive attitudes (*P* < 0.001), higher subjective norms (*P* < 0.001), and greater Alzheimer’s knowledge (*P* < 0.01).
Rhee and Jang 2020	Quantitative, cross-sectional design	*N* = 97 KA aged 60 years and older (total: 499 of 5 Asian American subgroups Age and immigration information not reported discretely	Various community locations, cultural events in Austin, Texas	Survey: 2015 Asian American Quality of Life survey	KAs had the lowest rate of substitute decision-maker designation (8.2%) among the 5 AA groups. Across the 5 AA groups, ethnicity, time in the US (over 10 years), English proficiency, and acculturation significantly predicted substitute decision-maker designation (*P* < 0.05).
Park 2021	Quality improvement project (single-group pre- and post-test design)	*N* = 11 KA caregivers of older adult parents (total: 13 Asian Americans) Mean age: 40. 5 (total) Immigration information not reported	A church in urban New Jersy mainly composed of KA members	Survey: Engagement in ACP, changes in ACP actions, Program evaluation and feedback	1.5-hour training, including a 20-minute film, ACP concepts overview, and a step-by-step guide on using the Five Wishes AD for family ACP discussions were provided. Engagement in ACP increased by 23% from pretest to the 2-month follow-up. 31% of participants completed a new action at post-test after 2 months. The most common change was initiating a new ACP conversation with a parent.
Hong et al. 2022	Qualitative, exploratory design	*N* = 30 KAs aged 65 or older Mean age: 74.8 Mean years in US: 33.5 All first-generation immigrants	A community agency in the Midwest Metropolitan area	Interview: Awareness, Attitude, Perceived barriers, and Preference for ACP	Many participants had insufficient knowledge or misunderstanding about ADs. They wanted to reduce adult children’s decision-making burden but were concerned about noncompliance with ADs. They preferred natural dying and physician-initiated ACP conversations. Most viewed their adult children, particularly the emotionally closest one, as key in ACP conversations.
Lee et al. 2022a	Quantitative, cross-sectional design	*N* = 259 KAs aged 65 or older Mean age: 44.9 Mean years in US: 10.2 72% with limited English proficiency	Community organizations in two counties in Alabama	Survey: Health literacy, Hospice awareness, Social isolation, Threats to interpersonal safety, Willingness to discuss EOL care	Most participants were willing to discuss EOL care with their families (94%) and doctors (82%). Higher hospice awareness increased the likelihood of engaging in discussions with family and doctors (*P* < 0.001). Barriers to seeing a doctor due to cost and safety concerns reduced desire to discuss EOL care with family. (*P* < 0.001). More chronic conditions (*P* = 0.005) and higher social isolation (*P* = 0.01) were linked to less willingness to discuss EOL care with doctors.
Ha et al. 2023	Qualitative, descriptive design	*N* = 2 KA social workers (total 12 social workers in Korea and US) Age not reported	KA Daycare centers in California	Interview: Comparison of cultural norms, attitudes, and preferences for ACP among US, Korean, and KA communities	Social worker participants reported that many KA individuals do not have POA, or living will and rely on their families for EOL decision-making. Social worker participants reported KA individuals begin thinking about ACP after a death-related event, reflecting Korean culture’s focus on the present rather than the afterlife. Social worker participants observed generational differences in ACP acceptance among KAs.

*Note*. AA: Asian American, ACP: advanced care planning, AD: advanced directives, EOL: end-of-life, KA: Korean American, LST: life-sustaining treatment, N: number, NHW: non-Hispanic Whites, POA: power of attorney.

## Results

### General characteristics of the studies

Table 1 summarizes the characteristics of 49 studies. Since 2003, 23 Korea-based ICU studies were identified, with 14 conducted after the 2018 LST Act, which allows patients to withhold or withdraw life-sustaining treatment (WWLT). None collected data directly from patients. Instead, insights came from surrogate or staff reports and retrospective records. While Korean studies involved more sample diversity across patients, families, and providers than US studies, patients were generally critically ill older adults near EOL.

From the US, 26 studies (1996-present) focused mainly on first-generation, Korean-speaking older adults (Berkman and Ko 2010; Dobbs et al. 2015; Frank et al. 1998; Hong et al. 2019, 2022; Jang et al. 2010; Ko and Berkman 2010, 2012; Ko and Lee 2010; Kwak and Salmon 2007; Park 2021) recruited in communities. Terminology for the population varied; most studies used the term KAs; few used Korean immigrants (Ko et al. 2013) or KA immigrants (Berkman and Ko 2009; Lee et al. 2022a). Most studies were conducted in community settings in metropolitan areas and regions with high KA densities. None took place in hospitals. Exploration of providers’ perspectives was limited, with only one study including social workers (Ha et al. 2023).

### Thematic synthesis of the studies

**Figure 2.**
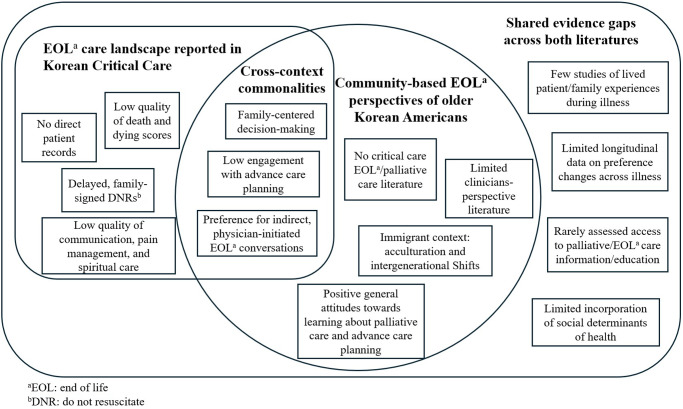
Mapping across Korean ICU and community-based Korean American literatures.

We identified four themes, each comprising commonalities and setting-specific patterns. Figure 2 maps scopes, gaps, and contextual features across both literatures. No US-based KA studies included ICU settings. Both bodies of literature shared elements, including family-centered decision-making, reluctance to engage in advance care planning (ACP), and a preference for physician-initiated EOL conversations. Korean ICU-based studies described high-acuity, near-death situations where the quality of dying was rated as inadequate. Conversely, KA studies elicited the perspectives of community-dwelling older adults through hypothetical scenarios, asking what they would prefer if they became ill. Differences in clinical context, legal structures, and social determinants alongside shared cultural traits shaped attitudes, knowledge, and perspectives.

### EOL critical care

#### Quality of EOL care in Korean ICUs

Three cross-sectional studies evaluated the QODD in Korean ICUs using surrogate reports from family members (Choi et al. 2019) or staff (Cho et al. 2019; Lee et al. 2022a). Across studies, overall QODD scores were low compared to reports from the US or Netherlands (Gerritsen et al. 2017) and hospice patients, with poor ratings in pain control, emotional support, and dignity (Cho et al. 2019; Choi et al. 2019; Lee et al. 2022a). However, scores were higher when patients transitioned to general wards before death and after the enactment of the 2018 LST Act, compared to the pre-enactment period (Cho et al. 2019; Choi et al. 2019; Lee et al. 2022a).

#### Evidence gap for critically ill KA populations

No US study examined KA patients in ICUs or other hospital settings. One community survey examining ACP intentions in a serious illness context (dementia) found that greater disease knowledge, more positive attitudes of ACP, and stronger perceived social norms (perceptions about what others would do and expect them to do) were associated with higher ACP intentions (Hong et al. 2019). However, this community-based design also shows gaps in how KA patients with illnesses navigate EOL or palliative care.

### Decision-making and communication

#### EOL decision-making and communication in Korean ICUs

Ten retrospective studies from Korean ICUs indirectly illustrated EOL decision-making patterns (Baek et al. 2016; Jang et al. 2023; Kim et al. 2016, 2024; Lee et al. 2020, 2008, 2021, 2024, 2019; Shin et al. 2014). Most do-not-resuscitate (DNR) orders, advance directives (AD), and Physician Orders for Life-Sustaining Treatment (POLST) were initiated by physicians (Lee et al. 2008; Shin et al. 2014) and signed by family members on behalf of patients (Baek et al. 2016; Jang et al. 2023; Kim et al. 2016; Lee et al. 2020). Completion rates for AD and POLST before or during ICU admission were consistently low, ranging from 0% to 15.7% (Jang et al. 2023; Kim et al. 2016; Lee et al. 2020, 2024; Shin et al. 2014).

Another consistent finding was that DNR or WWLT orders were commonly made very late, often to prevent anticipated, non-beneficial CPR when death became imminent (Jang et al. 2023; Kim et al. 2016; Lee et al. 2020, 2024; Shin et al. 2014). In three retrospective studies, conducted between 2008 and 2024, DNR or WWLT decisions were made within three days of patient death (Kim et al. 2024; Lee et al. 2008; Shin et al. 2014). A study examining DNR timing found that later DNR orders were associated with longer ICU stays and higher rates of WWLT (Baek et al. 2016). Another study similarly challenged the assumption that care intensity decreases with DNR or POLST, reporting longer ICU stays and higher mortality among patients with such orders (Lee et al. 2024).

The findings highlighted how POLST, designed for early, proactive EOL planning, was often used similarly to reactive approaches of WWLT. Qualitative studies interviewing nurses added contexts, indicating that discussions about life-sustaining treatment decisions rarely occur before patients become incapacitated and that both families and providers typically delay EOL conversations until patients’ conditions become critical (Chang et al. 2024; Koh et al. 2017).

Few prospective studies addressed the perceived quality of EOL communication in ICU-based Korean studies. One study found families’ perception of the quality of EOL communication to be low regarding patients’ condition, spiritual or religious beliefs, and treatment preferences (Jo et al. 2019). Another small-scale case study reported on the effectiveness of a multidisciplinary ICU family meeting protocol in facilitating information sharing between clinicians and families (Min et al. 2016).

#### Culturally sensitive EOL decision-making and communication preferences of KA populations

Many KA older adults responding to hypothetical scenarios reported strong preferences for indirect EOL communication to maintain hope when discussing severe illnesses (Berkman and Ko 2010; Blackhall et al. 2001; Kwak and Salmon 2007), with acknowledgments of the need for transparency to prepare for dying (Phipps et al. 2003) and concerns that disclosure could cause distress or hasten death. However, these US-based studies did not include clinical encounters, leaving a significant gap in understanding real-world care experiences and providers’ perspectives for the KA population within US healthcare settings.

#### Cross-context cultural insights: decision-making and communication

Family-centered decision-making with physician-led discussions was a common thread in both literatures. A Korea-based ICU study using focused ethnography explored the real-time decision-making experiences of families and showed that decisions often fell to the adult-child most financially or emotionally invested (Choi et al. 2023). Decisions were typically initiated by physicians, who explained the futility of treatment, and depended on families’ perceptions of the balance between the patient’s comfortable death and filial and financial obligations (Choi et al. 2023). Similarly, in US-based studies, older KA adults prioritized collective family decisions over individual autonomy (Berkman and Ko 2010; Blackhall et al. 1999; Frank et al. 1998; Hong et al. 2022; Ko and Berkman 2010, 2012; Kwak and Salmon 2007), and expected their adult children, the emotionally closest or eldest, to make care decisions and provide the utmost care to extend life (Frank et al. 1998; Hong et al. 2022; Ko and Berkman 2010, 2012; Kwak and Salmon 2007). KA participants also favored physician-initiated EOL conversations to ease emotional discomfort (Hong et al. 2022; Ko and Berkman 2010; Kwak and Salmon 2007) and preferred Korean providers (Berkman and Ko 2010; Kwak and Salmon 2007). AD completions were low among older KA adults, ranging from 0 to 18%, and often viewed as guidance for adult children rather than binding legal instructions upholding autonomy (Dobbs et al. 2015; Hong et al. 2019; Ko and Berkman 2010, 2012; Ko and Lee 2010; Kwak and Salmon 2007; Murphy et al. 1996). As KA studies primarily included first-generation older adults, interpretation is bounded by sample composition, leaving gaps on younger and US-born KAs and intergenerational dynamics.

### Perspectives and knowledge about EOL care

#### Perspectives and knowledge among Korean ICU family members

Five surveys of Korean ICU families assessed attitudes and perceptions regarding EOL care (Byun et al. 2003; Kim and Kang 2015; Kim and Tak 2021; Lee and Kang 2010; Oak and Kim 2020). Many family members supported WWLT for patient dignity (Byun et al. 2003; Lee and Kang 2010), while some preferred to be informed about the need for DNR in patients’ imminent death (Kim and Kang 2015). Knowledge of hospice care correlated positively with attitudes (Kim and Tak 2021; Oak and Kim 2020). However, these surveys only focused on family members and did not examine their education or access to information about AD or ACP.

#### Perspectives and knowledge among KA populations

Nine US-based studies reported generally low knowledge and awareness regarding AD, power of attorney, and ACP among KA participants (Dobbs et al. 2015; Ha et al. 2023; Hong et al. 2019, 2022; Jang et al. 2017; Ko and Berkman 2012; Ko and Lee 2010; Ko et al. 2013; Kwak and Salmon 2007). Although KA older adults favored low-intensity care and home death, many still preferred families to make EOL decisions for them (Blackhall et al. 1999, 1995; Frank et al. 1998; Ha et al. 2023; Ko and Berkman 2010, 2012; Kwak and Salmon 2007). While general, non-personal attitudes towards leveraging and learning about palliative care and ACP were positive, actual personal engagement remained limited (Berkman and Ko 2009; Jang et al. 2010; Ko and Berkman 2012; Lee et al. 2022a; Pan et al. 2015). Key factors that contributed to increased ACP intentions included greater knowledge and awareness, favorable subjective norms (how others behave and expect them to act), perceived better ability to discuss these topics, and recognition of benefits such as avoiding unwanted treatment (Hong et al. 2019; Jang et al. 2010; Ko and Lee 2010; Lee et al. 2022a). Health insurance coverage and higher educational attainment were also associated with more positive attitudes and awareness of AD (Dobbs et al. 2015; Kim and Foreman 2011). Stronger spiritual and religious beliefs were linked to greater engagement in EOL communication (Ko et al. 2013). Reported barriers included concerns about costs, social isolation, and chronic conditions (Lee et al. 2022a).

#### Cross-context cultural insights: perspectives and knowledge

Across both literatures, hesitancy around personal and family EOL planning was evident. In Korean ICUs, families advocated patient dignity yet preferred to defer DNR discussions until death was near (Kim and Kang 2015), whereas older KA adults preferred low-intensity care, peaceful death, but at times delegated decisions to adult children (Blackhall et al. 1999, 1995; Frank et al. 1998; Ha et al. 2023; Ko and Berkman 2010, 2012; Kwak and Salmon 2007). Although greater awareness correlated with positive attitudes toward EOL topics, studies rarely specified when, where, or how education, information access, and support were provided.

### Contextual and social determinants of EOL care

#### Familial and disease-related factors for Korean ICU patients

Two Korean ICU-based studies examined specific characteristics – comparing homeless and non-homeless individuals and cancer versus non-cancer patients (Kim et al. 2024; Lee et al. 2019). Although there was no difference in mortality, homeless ICU patients were reported to undergo more CPR procedures before death compared to non-homeless ICU patients in Korea, likely due to the absence of family members to make WWLT decisions on their behalf (Lee et al. 2019). In the study conducted post enactment of the LST Act, the type of diagnosed disease was found to influence EOL decision-making outcomes, with cancer patients more likely to make decisions for themselves compared to patients with neurological or infectious diseases (Kim et al. 2024). The results of these two studies highlighted specific aspects of social determinants and patient characteristics that pose challenges and influence EOL care decisions in the ICU, which warrant further investigation.

#### Sociocultural influences on EOL care preferences among KA populations

Sociocultural factors, including place of upbringing and acculturation, were key factors of EOL preferences among KA participants. Individuals with greater acculturation – through longer US residency, higher English proficiency, and younger age – tended to prefer transparency and autonomy, while less acculturated individuals often preferred traditional, family-centered, and indirect communication (Berkman and Ko 2009; Blackhall et al. 1995; Dobbs et al. 2015; Ha et al. 2023; Kim and Foreman 2011; Ko and Lee 2010; Rhee and Jang 2020). However, studies acknowledged limitations in capturing a wider range of reasons behind these attitudes (Dobbs et al. 2015; Ha et al. 2023; Jang et al. 2010) or shifting preferences alongside disease progression (Berkman and Ko 2010).

While acculturation was frequently addressed (Dobbs et al. 2015; Jang et al. 2010, 2017; Kim and Foreman 2011; Ko et al. 2012), many studies lacked detailed immigration-related information, such as generational status, citizenship, or English proficiency. Most studies primarily recruited first-generation older adults and referred to them as KAs with little differentiation between naturalized citizens and recent immigrants (Berkman and Ko 2010; Dobbs et al. 2015; Frank et al. 1998; Hong et al. 2019, 2022; Jang et al. 2010; Ko and Berkman 2010, 2012; Ko and Lee 2010; Kwak and Salmon 2007; Park 2021), limiting understanding of the nuanced effects of immigration backgrounds on EOL preferences. Six studies specified participants’ lengths of US residence (12 to 29 years) (Berkman and Ko 2009; Jang et al. 2010; Kim and Foreman 2011; Ko and Berkman 2012; Ko et al. 2012; Kwak and Salmon 2007), and one study reported that 72% of participants had limited English proficiency (Lee et al. 2022a), highlighting the need for more granular research on the sociocultural contexts of EOL.

#### Cross-context cultural insights: contextual and social determinants

Across both literature sets, EOL preferences and decisions are shaped by family structures, illness characteristics, and sociocultural factors, including generational shifts and language. In Korea, the relatively recent development of palliative care, reflected in the 2018 LST Act, coincided with low formal engagement in EOL planning (Choi et al. 2019; Kim et al. 2024). Among KAs, ongoing migration and limited English proficiency potentially add informational and linguistic hurdles (Lee et al. 2022a; U.S. Census Bureau 2022). These observations are hypothesis-generating rather than causal and are presented as contextual features that may help interpret cross-context cultural patterns.

## Discussion

### Research gaps and cross-cultural insights

This scoping review mapped the state of science to draw out transferable cultural insights in EOL and palliative care across Koreans in Korean ICUs and KAs in US community settings. Notably, no US-based studies included critically ill KAs in healthcare settings; likewise, prior reviews focused on community samples (Park and Hendrix 2018; Suk et al. 2021). Across both bodies of literature, primary emphases were on EOL decision-making, communication, ACP, attitudes, and knowledge. Symptom management, caregiving support, and spiritual/religious care were rarely addressed. Our aim was not to clinically and statistically compare across settings but to identify cross-context implications to inform culturally responsive EOL care. Common themes included family-centered decision-making – often led by adult-children – physician-initiated conversations, reluctance to disclose and discuss serious illnesses, and low engagement and knowledge of ACP/AD. Interpretations of each literature set are shaped and bounded by its socio-legal context and the acuity and proximity to death of the samples.

This cross-context cultural analysis illustrates how heritage-country evidence can inform research and practice for immigrant and minoritized populations with sparse domestic data. Korean ICU studies highlighting family-consented DNRs and late decisions made near death (Jang et al. 2023; Kim et al. 2016; Lee et al. 2020, 2024; Shin et al. 2014) offer hypotheses for research in KA populations. Future research on KA should investigate whether similar delayed decisions reflect cultural hesitation to discuss death or systemic barriers, and conduct formative research based on KA preferences to develop culturally tailored interventions. Such interventions could involve families earlier and shift indirect communication to upfront conversations by clinicians as readiness develops. Evaluating the effects of linguistic and cultural concordance between patients and providers can identify systemic factors that impact EOL planning and help create language-concordant care models. Comparative research across immigration generations, age groups, and Asian American subgroups may uncover important within-group differences for culturally sensitive EOL care.

### Rethinking metrics: access and strength-based perspectives

Existing US-based KA studies have primarily focused on knowledge, attitudes, or preferences toward EOL care, with little attention to actual access to palliative or EOL care services (Dobbs et al. 2015; Hong et al. 2019; Jang et al. 2010, 2017; Ko and Berkman 2010, 2012; Ko and Lee 2009, 2010; Ko et al. 2013; Kwak and Salmon 2007). Assessing access to palliative care is critical to avoid misattributing disparities to cultural preferences alone, which can risk framing cultural differences as deficiencies (Abu-Saad et al. 2021; Fogarty et al. 2018). Instead, strength-based approaches offer a more nuanced understanding by critically examining historic and systemic factors and emphasize community assets (Abu-Saad et al. 2021; Fogarty et al. 2018; McEwen et al. 2019). These distinctions are summarized in Table 4.

**Table 4.** Deficit-based and strength-based approaches

Dimension	Deficit-based approach	Strength-based approach
Focus	Identifying gaps, needs, and deficiencies	Identifying assets, capacities, and positive resources
Underlying assumption	Gaps from standards reflect knowledge or skill deficits	Cultural differences reflect genuine preferences or different social, historical, political, economic contexts
Goals	Develop interventions targeting the root causes of individual or community problems	Design interventions that leverage community assets and address social determinants
Example question	“What barriers prevent KA individuals from engaging in ACP?”	“How does family cohesion support KA individuals’ sense of dignity and comfort in making decisions?”

For example, AD completion was commonly used as an indicator of EOL preparedness, which may not fully account for cultural preferences, such as Koreans’ emphasis on relativistic and family-centered values (Dobbs et al. 2015; Hong et al. 2019; Jang et al. 2017; Ko and Berkman 2012; Ko and Lee 2010; Lee et al. 2020; Shin et al. 2014). Thus, focusing solely on this Western-centric metric may overlook culturally specific approaches, such as ongoing family communication as a valid alternative for EOL planning (McDermott and Selman 2018), underscoring the need for future research to develop more inclusive and culturally sensitive palliative care metrics in today’s increasingly multicultural world (Ntizimira et al. 2022).

### Methodological innovations for EOL research in ICU

A key limitation of ICU-based studies in Korea was the reliance on surrogate reports (Cho et al. 2019; Choi et al. 2019; Lee et al. 2022a) and medical records (Baek et al. 2016; Jang et al. 2023; Kim et al. 2016, 2024; Lee et al. 2020, 2008, 2021, 2024, 2019; Shin et al. 2014). Innovative methods are needed to capture experiences among patients facing frailty, cognitive decline, and communication difficulties. Recruiting ICU survivors, despite possible memory limitations, may help address the lack of direct patient experience in surrogate reports (van de Leur et al. 2004). Other innovative methods include observation of care revealing subtle aspects of patient experiences with minimal risk (Eriksson et al. 2010), ICU diaries offering longitudinal documentation (Sayde et al. 2020), and grey literature such as patients’ and families’ social media posts providing authentic narratives (Taylor and Pagliari 2018). These approaches can expand our understanding of actual palliative care delivery in critical care, promoting more empathetic strategies while minimizing patient burden.

### Practice and policy implications for EOL care in Korea

Cultural, social, and legal components shape healthcare practices. The 2018 LST Act in Korea authorized patients to refuse futile life-sustaining treatments through AD or, by the will of family members (Lee et al. 2021), which may have reinforced families as primary decision-makers (Choi et al. 2023) and posed challenges for patients without legal immediate family (Lee et al. 2019). Criteria for POLST orders – confirmation by two physicians of patient status, presence of hospital ethics committees, and availability of next-of-kin (Heo et al. 2022) – may further limit decision-making options for those without such supports. Expanding the legal definition of “next-of-kin” and improving support are crucial for vulnerable populations. In addition, EOL care disparities exist based on diagnosis (Kim et al. 2024), reflecting how cancer-centered palliative care models have long dominated resources (Kim and Hong 2016), leaving other patients with limited access to timely palliative referrals or discussions. To ensure fair access, palliative care in Korea should broaden its focus beyond cancer and develop advocacy for socioeconomically disadvantaged, unbefriended, and homeless individuals (Jang et al. 2022; Park et al. 2024).

### Practice and policy implications for EOL care in the US

While disparities in Korea stem from a relatively homogeneous population, KA individuals in the US exhibit substantial internal diversity by immigration status, generation, and age cohorts. How US-borns, naturalized citizens, and recently arrived immigrants may differ in their cultural identities is insufficiently captured in research and health systems, often assuming homogeneous preferences. Integrating disaggregated data is crucial for capturing both within- and between-group differences more effectively. To promote implementation, these practices could be tied to hospital quality ratings and billing codes and inform more tailored EOL and palliative care delivery.

### Limitations and implications for future research

This review is limited by the scope of Korean ICU-based studies, which may not reflect the broader Korean population or individuals with pre-ICU opportunities to access palliative care. As many patients are more cognitively intact before ICU admission, future studies should target pre-ICU patient preferences and interventions to examine the trajectories and influence on EOL outcomes in ICU settings. Additionally, we included studies with subsamples of the target population that reported relevant discrete findings, as this review aimed to broadly map the field for cultural insights. Therefore, the findings are exploratory in identifying cultural patterns and should not be considered generalizable or definitive conclusions.

## Conclusion

This scoping review examined EOL care among critically ill Koreans in Korea and KA communities in the US. Findings highlight the need to explore EOL perspectives and disparities shaped by sociocultural, legal, and systemic factors and call attention to addressing immigration, generational, and acculturation-related differences within the KA populations with critical illnesses. While gaps exist regarding critically ill KA individuals, Korean ICU literature provides insights to inform culturally informed care. Culturally tailored strategies, building on the strengths of family-centered communities, are needed in EOL and palliative care research for KA populations. This cross-contextual perspective and the review design can inform research and practice for other minoritized or immigrant groups.

## Appendix A

**Table:** Bibliographic database search strategies (search date: 07/02/2024) 1. PubMed 1) Korea

	Query	Results
4	((((((((“Hospice Care”[Mesh]) OR “Terminal Care”[Mesh]) OR “Palliative Care”[Mesh]) OR “Hospice and Palliative Care Nursing”[Mesh]) OR “Palliative Medicine”[Mesh] OR “Advance Care Planning”[Mesh]) OR “Advance Directives”[Mesh] OR “end of life care”[TIAB] OR “terminal care”[TIAB] OR “palliative care”[TIAB] OR “hospice care”[TIAB]) OR “advance care planning”[TIAB] OR “advance directives”[TIAB] OR “goals of care” [TIAB] OR “care goals” [TIAB] OR “living will”[TIAB]) AND ((“Critical Care”[Mesh]) OR “Intensive Care Units”[Mesh] OR “critical care”[TIAB] OR “intensive care”[TIAB])) AND (“Asian”[Mesh] OR “East Asian People”[Mesh] OR “Korea*” OR “Asia*”)	139
3	(”Asian”[Mesh] OR “East Asian People”[Mesh] OR “Korea*” [TW] OR “Asia*” [TW])	410,751
2	(“Critical Care”[Mesh]) OR “Intensive Care Units”[Mesh] OR “critical care”[TIAB] OR “intensive care”[TIAB]	298,484
1	((((((“Hospice Care”[Mesh]) OR “Terminal Care”[Mesh]) OR “Palliative Care”d[Mesh]) OR “Hospice and Palliative Care Nursing”[Mesh]) OR “Palliative Medicine”[Mesh] OR “Advance Care Planning”[Mesh]) OR “Advance Directives”[Mesh] OR “end of life care”[TIAB] OR “terminal care”[TIAB] OR “palliative care”[TIAB] OR “hospice care”[TIAB]) OR “advance care planning”[TIAB] OR “advance directives”[TIAB] OR “goals of care” [TIAB] OR “care goals” [TIAB] OR “living will”[TIAB]	137,766

**Table:** 2) US

	Query	Results
4	(((((((“Hospice Care”[Mesh]) OR “Terminal Care”[Mesh]) OR “Palliative Care”[Mesh]) OR “Hospice and Palliative Care Nursing”[Mesh]) OR “Palliative Medicine”[Mesh] OR “Advance Care Planning”[Mesh]) OR “Advance Directives”[Mesh] OR “end of life care”[TIAB] OR “terminal care”[TIAB] OR “palliative care”[TIAB] OR “hospice care”[TIAB]) OR “advance care planning”[TIAB] OR “advance directives”[TIAB] OR “goals of care” [TIAB] OR “care goals” [TIAB] OR “living will”[TIAB]) AND (“Asian”[Mesh] OR “East Asian People”[Mesh] OR “Korea*” [TW] OR “Asia*” [TW]) NOT ((africa[MESH] OR asia[MESH] OR australia[MESH] OR canada[MESH] OR central america[mesh] OR europe[MESH] OR south america[MESH]) NOT (north america[MESH:NOEXP] OR united states[MESH]))	852
3	NOT ((Africa[MESH] OR asia[MESH] OR australia[MESH] OR canada[MESH] OR central america[mesh] OR europe[MESH] OR south america[MESH]) NOT (north america[MESH:NOEXP] OR united states[MESH]))	
2	“Asian”[Mesh] OR “East Asian People”[Mesh] OR “Korea*” [TW] OR “Asia*” [TW]	410,751
1	((((((“Hospice Care”[Mesh]) OR “Terminal Care”[Mesh]) OR “Palliative Care”[Mesh]) OR “Hospice and Palliative Care Nursing”[Mesh]) OR “Palliative Medicine”[Mesh] OR “Advance Care Planning”[Mesh]) OR “Advance Directives”[Mesh] OR “end of life care”[TIAB] OR “terminal care”[TIAB] OR “palliative care”[TIAB] OR “hospice care”[TIAB]) OR “advance care planning”[TIAB] OR “advance directives”[TIAB] OR “goals of care” [TIAB] OR “care goals” [TIAB] OR “living will”[TIAB]	137,766

**Table:** 2. CINAHL (Cumulated Index to Nursing and Allied Health Literature) 1) Korea

	Query	Results
4	(S1 AND S2 AND S3)	101
3	(MH “Korean Americans”) OR (MH “Koreans”) OR (MH “South Korea”) OR (MH “Asian Americans +”) OR (MH “East Asian Americans +”) OR TI (Korea* OR Asia*) OR AB (Korea* OR Asia*)	85,578
2	(MH “Intensive Care Units +”) OR (MH “Critical Care +”) OR (MH “Critical Care Nursing +”) OR TI(“intensive care” OR “critical care”) OR AB(“intensive care” OR “critical care”)	156,868
1	(MH “Terminal Care +”) OR (MH “Hospice Care”) OR (MH “Palliative Care”) OR (MH “Advance Care Planning”) OR (MH “Palliative Care Nursing”) OR (MH “Palliative Medicine”) OR TI(“end of life care” OR “terminal care” OR “palliative care” OR “hospice care” OR “advance care planning” OR “advance directives” OR ‘goals of care’ OR ‘care goals’ OR ‘living will’) OR AB(“end of life” OR “palliative care” OR “terminal care” OR ‘hospice care’ OR “advance care planning” OR “advance directives” OR ‘goals of care’ OR ‘care goals’ OR ‘living will’)	93,805

**Table:** 2) US

	Query	Results
4	(S1 AND S2 ) Narrow by Subject Geographic: – US	456
3	(S1 AND S2 )	1,074
2	(MH “Korean Americans”) OR (MH “Koreans”) OR (MH “South Korea”) OR (MH “Asian Americans +”) OR (MH “East Asian Americans +”) OR TI (Korea* OR Asia*) OR AB (Korea* OR Asia*)	81,032
1	(MH “Terminal Care +”) OR (MH “Hospice Care”) OR (MH “Palliative Care”) OR (MH “Advance Care Planning”) OR (MH “Palliative Care Nursing”) OR (MH “Palliative Medicine”) OR TI(“end of life” OR “terminal care” OR “palliative care” OR ‘hospice care’ OR “advance care planning” OR “advance directives” OR ‘goals of care’ OR ‘care goals’ OR ‘living will’) OR AB(“end of life” OR “palliative care” OR “terminal care” OR ‘hospice care’ OR “advance care planning” OR “advance directives” OR ‘goals of care’ OR ‘care goals’ OR ‘living will’)	93,650

**Table:** 3. EMBASE 1) Korea

	Query	Results
4	#1 AND #2 AND #3	484
3	‘korean (people)’/exp OR ‘korean american’/exp OR ‘asian american’/exp OR ‘asian’/exp OR (korea* OR asia*):ti,ab,kw	666,664
2	‘intensive care nursing’/exp OR ‘intensive care’/exp OR ‘intensive care unit’/exp OR ((intensive OR critical) NEAR/2 care):ti,ab,kw	1,231,906
1	“palliative therapy”/exp OR “palliative nursing”/exp OR “hospice care”/exp OR “terminal care”/exp OR “advance care planning”/exp OR “living will”/exp OR (‘end of life care’ OR ‘terminal care’ OR ‘palliative care’ OR “hospice care” OR “advance care planning” OR “advance directives” OR “goals of care” OR “care goals” OR “living will”):ti,ab,kw	240,401

**Table:** 2) US

	Query	Results
4	#1 AND #2 AND #3	2434
3	NOT ((‘Africa’/exp OR ‘Asia’/exp OR ‘Australia’/exp OR ‘Canada’/exp OR ‘Central America’/exp OR ‘Europe’/exp OR ‘South America’/exp) NOT (‘North America’/de OR ‘United States’/exp))	
3	#1 AND #2	4,080
2	‘korean (people)’/exp OR ‘korean american’/exp OR ‘asian american’/exp OR ‘asian’/exp OR (korea* OR asia*):ti,ab,kw	666,664
1	“palliative therapy”/exp OR “palliative nursing”/exp OR “hospice care”/exp OR “terminal care”/exp OR “advance care planning”/exp OR “living will”/exp OR (‘end of life care’ OR ‘terminal care’ OR ‘palliative care’ OR “hospice care” OR “advance care planning” OR “advance directives” OR “goals of care” OR “care goals” OR “living will”):ti,ab,kw	240,401

**Table:** 4. Web of Science 1) Korea

	Query	Results
4	#3 AND #2 AND #1	128
3	TS = (“Asia*” OR “Korea*”)	741,407
2	TS = (“intensive care” OR “critical care”)	269,384
1	TS = (“end of life care” OR “palliative care” OR “terminal care” OR ‘hospice care’ OR “advance care planning” OR “advance directives” OR ‘goals of care’ OR ‘care goals’ OR ‘living will’)	78,922

**Table:** 2) US

	Query	Results
4	#2 AND #1 AND US (Countries/Regions)	408
3	#2 AND #1	1,256
2	TS = (“Asia*” OR “Korea*”)	741,407
1	TS = (“end of life care” OR “palliative care” OR “terminal care” OR ‘hospice care’ OR “advance care planning” OR “advance directives” OR ‘goals of care’ OR ‘care goals’ OR “living will”)	78,922

**Table:** 5. KCI – Korea Citation Index

	Query	Results
7	#3 AND #6	91
6	#4 OR #5	5,853
5*	TS = (중환자 OR 중환자실 OR 중환자간호)	3,440
4	TS = ((“Critical Care” OR “Intensive Care Units” OR “intensive care”) )	3,315
3	#1 OR #2	1,405
2*	TS = (완화의료 OR 생애말기 OR 호스피스)	667
1	TS = (“end of life care” OR “Hospice Care” OR “Terminal Care” OR “Palliative Care” OR “advance care planning” OR “advance directives” OR ‘goals of care’ OR ‘care goals’ OR ‘living will’ )	1,151

*Translation.

#2 = #1 in Korean.

#5 = #4 in Korean.

**Table:** 6. Global Index Medicus – West Pacific

Query		Results
4	#1 AND #2 AND 3 (tw:(“Asia*” OR “Korea*”) AND tw:(“Critical Care” OR “Intensive Care Units” OR “intensive care”) AND tw:(“end of life care” OR “Hospice Care” OR “Terminal Care” OR “Palliative Care” OR “advance care planning” OR “advance directives” OR ‘goals of care’ OR ‘care goals’ OR ‘living will’) AND ( collection_gim:(“WPRIM”))	43
3	tw:(“Asia*” OR “Korea*”)	38,795
2	tw:(“Critical Care” OR “Intensive Care Units” OR “intensive care”)	9,717
1	tw:(“end of life care” OR “Hospice Care” OR “Terminal Care” OR “Palliative Care” OR “advance care planning” OR “advance directives” OR ‘goals of care’ OR ‘care goals’ OR ‘living will’)	34,629

**Table:** 7. KoreanMed

Query		Results
3	#1 AND #2	32
2	“Critical care”[TIAB] OR “Intensive care”[TIAB] OR “Intensive Care Unit”[TIAB]	4,056
1	“end of life care”[TIAB] OR “Hospice Care”[TIAB] OR “Terminal Care”[TIAB] OR “Palliative Care”[TIAB] OR “Advance care planning”[TIAB] OR “Advance directives”[TIAB] OR “goals of care”[TIAB] OR ‘care goals’[TIAB] OR “living will”[TIAB]	516

Databases 5–7 were searched only for Korean-based studies. For database 5 and 7, search was conducted without including “Koreans” or “Asians” in the search queries, given that these databases were based in Korea.

## Appendix B

**Table:** Preferred Reporting Items for Systematic reviews and Meta-Analyses extension for Scoping Reviews (PRISMA-ScR) Checklist

SECTION	ITEM	PRISMA-ScR CHECKLIST ITEM	REPORTED ON PAGE #
**TITLE**			
Title	1	Identify the report as a scoping review.	Pg.1
**ABSTRACT**			
Structured summary	2	Provide a structured summary that includes (as applicable): background, objectives, eligibility criteria, sources of evidence, charting methods, results, and conclusions that relate to the review questions and objectives.	Pg.2
**INTRODUCTION**			
Rationale	3	Describe the rationale for the review in the context of what is already known. Explain why the review questions/objectives lend themselves to a scoping review approach.	Pg.4
Objectives	4	Provide an explicit statement of the questions and objectives being addressed with reference to their key elements (e.g., population or participants, concepts, and context) or other relevant key elements used to conceptualize the review questions and/or objectives.	Pg.5
**METHODS**			
Protocol and registration	5	Indicate whether a review protocol exists; state if and where it can be accessed (e.g., a Web address); and if available, provide registration information, including the registration number.	N/A
Eligibility criteria	6	Specify characteristics of the sources of evidence used as eligibility criteria (e.g., years considered, language, and publication status), and provide a rationale.	Pg.5–6
Information sources*	7	Describe all information sources in the search (e.g., databases with dates of coverage and contact with authors to identify additional sources), as well as the date the most recent search was executed.	Pg.5–6, Appendix A
Search	8	Present the full electronic search strategy for at least one database, including any limits used, such that it could be repeated.	Appendix A
Selection of sources of evidence†	9	State the process for selecting sources of evidence (i.e., screening and eligibility) included in the scoping review.	Pg.5–6
Data charting process‡	10	Describe the methods of charting data from the included sources of evidence (e.g., calibrated forms or forms that have been tested by the team before their use, and whether data charting was done independently or in duplicate) and any processes for obtaining and confirming data from investigators.	Pg.6
Data items	11	List and define all variables for which data were sought and any assumptions and simplifications made.	Pg.5–6
Critical appraisal of individual sources of evidence§	12	If done, provide a rationale for conducting a critical appraisal of included sources of evidence; describe the methods used and how this information was used in any data synthesis (if appropriate).	N/A
Synthesis of results	13	Describe the methods of handling and summarizing the data that were charted.	Pg.6–7
**RESULTS**			
Selection of sources of evidence	14	Give the number of sources of evidence screened, assessed for eligibility, and included in the review, with reasons for exclusions at each stage, ideally using a flow diagram.	Pg.5–6
Characteristics of sources of evidence	15	For each source of evidence, present characteristics for which data were charted and provide the citations.	Pg.11
Critical appraisal within sources of evidence	16	If done, present data on critical appraisal of included sources of evidence (see item 12).	N/A
Results of individual sources of evidence	17	For each included source of evidence, present the relevant data that were charted that relate to the review questions and objectives.	Pg.6–13
Synthesis of results	18	Summarize and/or present the charting results as they relate to the review questions and objectives.	Pg.6–13
**DISCUSSION**			
Summary of evidence	19	Summarize the main results (including an overview of concepts, themes, and types of evidence available), link to the review questions and objectives, and consider the relevance to key groups.	Pg.13–17
Limitations	20	Discuss the limitations of the scoping review process.	Pg.17
Conclusions	21	Provide a general interpretation of the results with respect to the review questions and objectives, as well as potential implications and/or next steps.	Pg.17
**FUNDING**			
Funding	22	Describe sources of funding for the included sources of evidence, as well as sources of funding for the scoping review. Describe the role of the funders of the scoping review.	Pg.17

JBI = Joanna Briggs Institute; PRISMA-ScR = Preferred Reporting Items for Systematic reviews and Meta-Analyses extension for Scoping Reviews.

* Where *sources of evidence* (see second footnote) are compiled from, such as bibliographic databases, social media platforms, and Web sites.

† A more inclusive/heterogeneous term used to account for the different types of evidence or data sources (e.g., quantitative and/or qualitative research, expert opinion, and policy documents) that may be eligible in a scoping review as opposed to only studies. This is not to be confused with *information sources* (see first footnote).

‡ The frameworks by Arksey and O’Malley (6) and Levac and colleagues (7) and the JBI guidance (4, 5) refer to the process of data extraction in a scoping review as data charting.

§ The process of systematically examining research evidence to assess its validity, results, and relevance before using it to inform a decision. This term is used for items 12 and 19 instead of “risk of bias” (which is more applicable to systematic reviews of interventions) to include and acknowledge the various sources of evidence that may be used in a scoping review (e.g., quantitative and/or qualitative research, expert opinion, and policy document).

*From:* Tricco AC, Lillie E, Zarin W, O’Brien KK, Colquhoun H, Levac D, et al. PRISMA Extension for Scoping Reviews (PRISMAScR): Checklist and Explanation. Ann Intern Med. 2018;169:467–473. doi: 10.7326/M18-0850.
